# Efficacy of endotracheal tube clamping to prevent positive airways pressure loss and pressure behavior after reconnection: a bench study

**DOI:** 10.1186/s40635-023-00519-1

**Published:** 2023-06-30

**Authors:** Enrico Bulleri, Cristian Fusi, Stefano Bambi, Luigi Pisani, Alice Galesi, Enrico Rizzello, Alberto Lucchini, Paolo Merlani, Alberto Pagnamenta

**Affiliations:** 1 Intensive Care Unit, Department of Anaesthesiology, Emergency and Intensive Care Medicine (DAEICM), Ente Ospedaliero Cantonale (EOC), Via Tesserete, 46, 6900 Lugano, Switzerland; 2 Department of Health Sciences, University of Florence, Florence, Italy; 3 Mahidol Oxford Tropical Medicine Research Unit, Bangkok, Thailand; 4 Department of Intensive Care and Anesthesia, Fondazione Poliambulanza di Brescia, Brescia, Italy; 5 Intensive Care Unit, Miulli Regional Hospital, Acquaviva delle Fonti, Italy; 6 Department of Emergency and Intensive Care, Fondazione IRCCS San Gerardo dei Tintori Monza, Monza, Italy; 7 Department of Anesthesiology, Pharmacology and Intensive Care, Geneva University Hospitals, Geneva, Switzerland; 8 Clinical Trial Unit, EOC, Lugano, Switzerland; 9 Division of Pneumology, Geneva University Hospitals, Geneva, Switzerland; 10 University of Milano-Bicocca, Milano, Italy

**Keywords:** ARDS, PEEP, Derecruitment

## Abstract

**Background:**

Endotracheal tube (ETT) clamping before disconnecting the patient from the mechanical ventilator is routinely performed in patients with acute respiratory distress syndrome (ARDS) to minimize alveolar de-recruitment. Clinical data on the effects of ETT clamping are lacking, and bench data are sparse. We aimed to evaluate the effects of three different types of clamps applied to ETTs of different sizes at different clamping moments during the respiratory cycle and in addition to assess pressure behavior following reconnection to the ventilator after a clamping maneuver.

**Methods:**

A mechanical ventilator was connected to an ASL 5000 lung simulator using an ARDS simulated condition. Airway pressures and lung volumes were measured at three time points (5 s, 15 s and 30 s) after disconnection from the ventilator with different clamps (Klemmer, Chest-Tube and ECMO) on different ETT sizes (internal diameter of 6, 7 and 8 mm) at different clamping moments (end-expiration, end-inspiration and end-inspiration with tidal volume halved). In addition, we recorded airway pressures after reconnection to the ventilator. Pressures and volumes were compared among different clamps, different ETT-sizes and the different moments of clamp during the respiratory cycle.

**Results:**

The efficacy of clamping depended on the type of clamp, the duration of clamping, the size of the ETT and the clamping moment. With an ETT ID 6 mm all clamps showed similar pressure and volume results. With an ETT ID 7 and 8 mm only the ECMO clamp was effective in maintaining stable pressure and volume in the respiratory system during disconnection at all observation times. Clamping with Klemmer and Chest-Tube at end inspiration and at end inspiration with halved tidal volume was more efficient than clamping at end expiration (*p* < 0.03). After reconnection to the mechanical ventilator, end-inspiratory clamping generated higher alveolar pressures as compared with end-inspiratory clamping with halved tidal volume (*p* < 0.001).

**Conclusions:**

ECMO was the most effective in preventing significant airway pressure and volume loss independently from tube size and clamp duration. Our findings support the use of ECMO clamp and clamping at end-expiration. ETT clamping at end-inspiration with tidal volume halved could minimize the risk of generating high alveolar pressures following reconnection to the ventilator and loss of airway pressure under PEEP.

## Introduction

In normal lungs alveoli reduce their size during expiration without collapsing, due to the presence of phospholipoproteic surfactant, that covering alveolar epithelium stabilizes respiratory units [1]. In patients with acute respiratory distress syndrome (ARDS), the coexistence of surfactant depletion, alveolo-capillary barrier damage, leukocytes migration to intra-alveolar spaces, pulmonary edema and gravitational forces promotes alveolar collapse during expiration, leading to a significant reduction of the functional residual capacity (FRC) [2]. The application of positive end-expiratory pressure (PEEP) counterbalances this phenomenon by maintaining alveoli open and stabilized. The main benefits of PEEP in this setting are a reduction in atelectrauma and in intrapulmonary shunt [3, 4].

The loss of PEEP is deemed particularly dangerous in ARDS patients [5, 6]. Disconnecting the patient from the mechanical ventilator is a frequently necessary procedure, to allow maneuvers such as replacement of ventilator circuit components. In a porcine model of acute lung injury, lung collapse occurs within a few seconds after positive airway pressure (Paw) loss and changes in lung density, in aerated tissue and atelectasia occur mainly within the first 4 s following disconnection from the ventilator [7]. In experimental ARDS, ventilator disconnection leads to a FRC reduction of more than 50% as assessed by electric impedance tomography [8] and abrupt pressure drop is associated with pulmonary edema, impaired oxygenation and increased pulmonary vascular resistance [5].

Endotracheal tube (ETT) clamping, performed seconds before disconnecting the patient from the mechanical ventilator, has become a well-established practice in the ICU setting to prevent alveolar collapse [9–11]. To the best of our knowledge, clinical data of the effects of clamping on pressure and lung volumes before disconnecting the patient from the ventilator are still lacking. A very recently published bench study found that the effects of clamping at the end of expiration on airway pressures and volumes depend on the type of clamp, the type of ETT and the duration of disconnection [12]. However, data on the effects of clamping in relation to ETT size and to the clamping moment during the respiratory cycle are still lacking. Moreover, airway pressure behavior following reconnection to the ventilator after an ETT clamping is still unknown.

We therefore conducted a bench study aiming at evaluating the effects of three different types of clamps on ETT of different sizes and at different clamping moments during the respiratory cycle. Furthermore, we assessed the impact on airway pressure following reconnection to the mechanical ventilator.

## Materials and methods

This study was conducted in the research and development laboratory of Air Liquid Medical Systems (Bovezzo, Italy). A mechanical ventilator (Monnal T 75: Air Liquide Medical Systems, Antony, France) was connected to ETTs with internal diameter (ID) of 6, 7 and 8 mm (Shiley, Covidien llc, Mansfield, USA). For each experiment the ETT was connected to a high-fidelity simulator of the respiratory system (ASL 5000, Ingmar inc., Pittsburgh, USA) via a 1.6 m long 22 mm smoothbore circle breathing circuit (Intersurgical, Wokingham, UK). We used an analogue manometer (VBM Medizintechnik GmbH, Sulz am Neckar, Germany) to set optimal ETTs cuff pressure. The ventilator settings were as follows: volume-controlled ventilation (VCV), tidal volume (TV) 360 ml; PEEP 10 cm H_2_O; respiratory rate (RR) 20 breaths per minute; inspiration time 1 s.

**Fig. 1:**
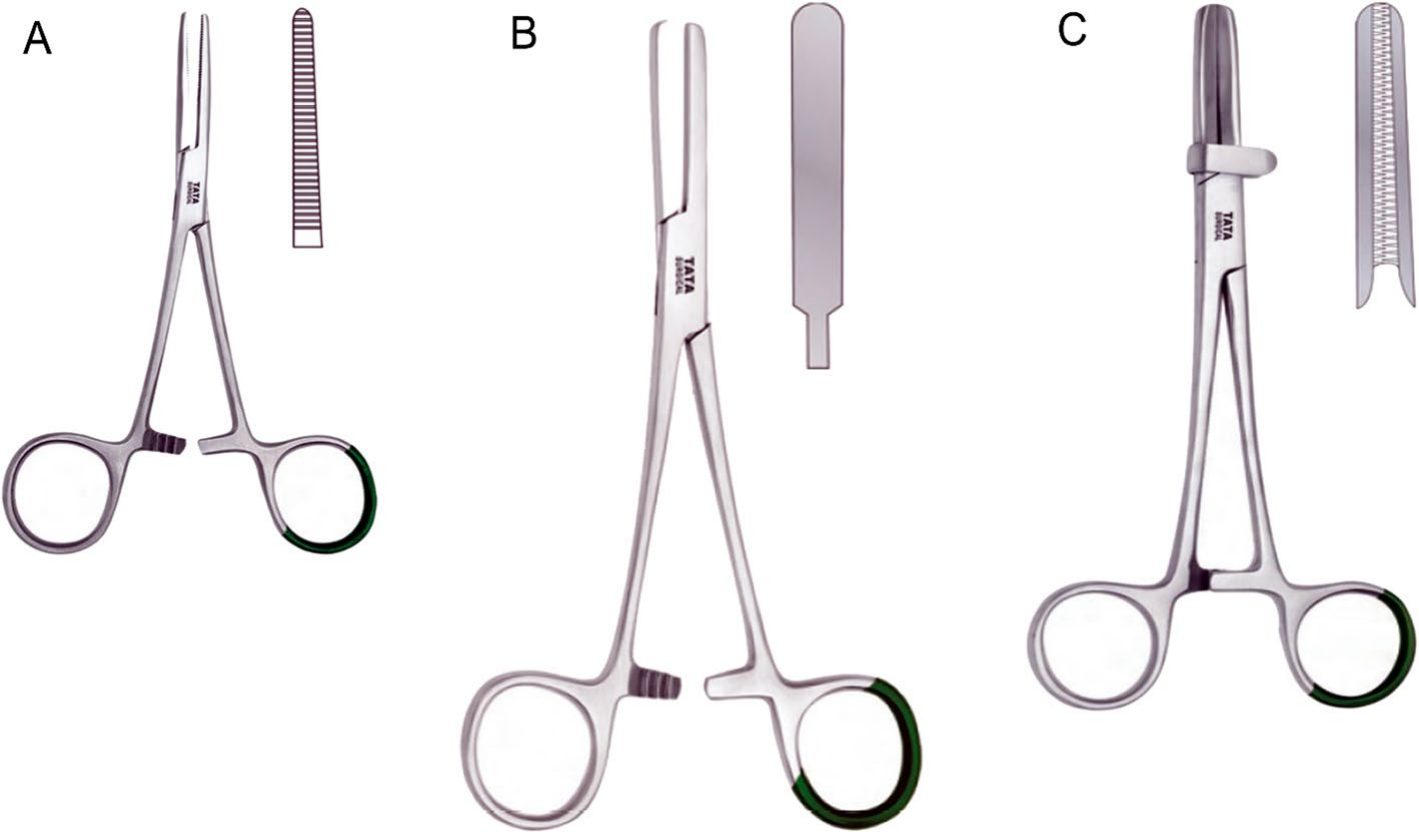
Type of clamp tested. Rochester-Pèan forceps straight 14 cm (**A**) thus named “Klemmer”, tubing Clamp Forceps 20 cm (**B**) thus named “Chest-Tube” and Tubing Clamp Forceps 18 cm (**C**) thus named “ECMO”

Three different types of clamps were tested:

1. Rochester-Pèan Forceps straight 14 cm (Promedical AG, Glarus, Switzerland); thus named “Klemmer”; (Fig. 1A).
2. Tubing Clamp Forceps 20 cm (Promedical AG, Glarus, Switzerland); thus named “Chest-Tube”; (Fig. 1B).
3. Tubing Clamp Forceps 18 cm (Promedical AG, Glarus, Switzerland), thus named “ECMO”, (Fig. 1C).

Efficacy of the clamping maneuver was defined as the ability to maintain an alveolar pressure during disconnection of at least 10 cm H_2_O. A Palv < 10 cm H_2_O and lung volume < 300 ml were considered critical values for clamping failure.

The lung model was set in passive ‘ARDS like’ conditions with a respiratory system compliance (Crs) of 30 ml/cm H_2_O and resistance of 8 cm H_2_O/L/sec. The model was connected via a standard ethernet connection to a personal computer containing the host software (ASL 5000 version 3.6). The data provided by the lung model were airway pressure (Paw) and lung volume (Vol); Paw and Vol were recorded immediately before disconnection and during tube clamping at 5, 15 and 30 s after disconnection.

Three different clamping moments during the respiratory cycle were tested:

1. End-expiration: clamping carried out during an end-expiratory occlusion.
2. End-inspiration: clamping carried out during an end-inspiratory occlusion.
3. 50%-end-inspiration: clamping carried out during an end-inspiratory occlusion, but with tidal volume halved (TV = 180 ml).

### Experimental protocol

#### Effects of clamping on pressure and volume

**Fig. 2:**
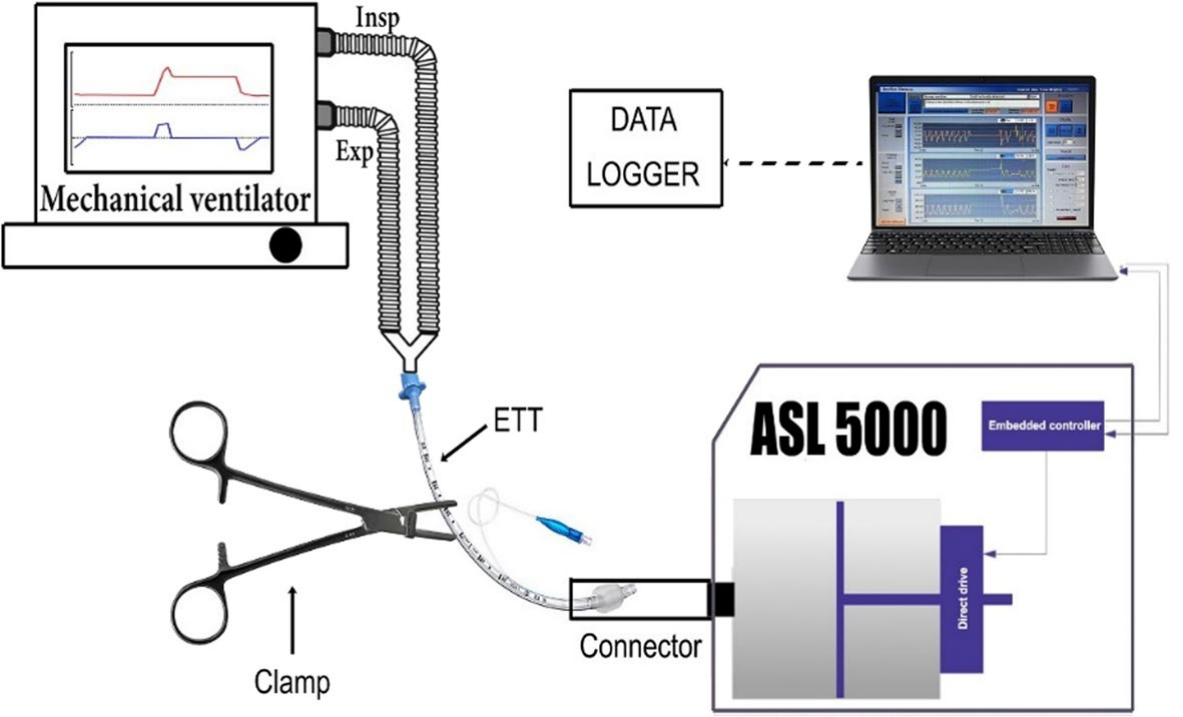
Schematic representation of the circuits used to perform the tightening tests. After clamping the tube the ventilator was removed, pressure and volume were read on datalogger. ETT, endotracheal tube; Exp, expiratory limb of the breathing circuit; Insp, inspiratory limb of the breathing circuit

- Step 1: the ETT was inserted into the connector and the cuff inflated at 30 cm H_2_O. The optimal anchorage of the ETT in the connector was checked manually.
- Step 2: the presence of air leaks within the respiratory system was evaluated after the ventilator, tube and lung model were connected. The ventilator was set as previously mentioned. To assess the tightness of the system, four respiratory cycles were delivered before performing a 15-s inspiratory occlusion. We excluded air leaks if the Paw was maintained stable during the inspiratory occlusion.
- Step 3: the clamp was applied perpendicular to the ETT and positioned about 5 cm from the ventilator side connector, maximizing the number of teeth tightening on ETT (Fig. 2). After clamping, the ETT was disconnected from the ventilator. During disconnection, pressures and volumes were recorded at 5, 15 and 30 s.

The three described steps were repeated for the three ETT (ID 6 mm, 7 mm and 8 mm), the three clamps (Klemmer; Chest-Tube; ECMO) and the three clamping moments during the respiratory cycle (end-expiration, end-inspiration; 50%-end-inspiration). Each measurement was repeated three times.

#### Effects of reconnection to the ventilator on plateau and driving pressure after a clamping maneuver

- Step 1 and step 2: as previously described in part A.
- Step 3: clamping was performed at end-inspiration and at 50%-end-inspiration using only ETT of 8 mm ID. After clamping, the ventilator was disconnected for 10 s and then reconnected, waiting for 2 insufflations before removing the clamp during the expiratory phase of the respiratory cycle. Paw and driving pressure of the first post-reconnection insufflation were recorded. Driving pressure (DP) is defined as plateau pressure minus PEEP. The maneuver was repeated with different clamps (Klemmer, Chest-Tube, ECMO). Each measure was repeated six times.

### Statistical analysis

Pressure and volume data were presented as median with the 1st and 3th quartile. The effects of different clamps (Klemmer, Chest-Tube, ECMO) on pressures and volumes were assessed by maintaining fixed the clamping moment during the respiratory cycle (end-expiration; end-inspiration; 50%-end-inspiration). The effects of different clamping moments during the respiratory cycle (end-expiration; end-inspiration; 50%-end-inspiration) were assessed by maintaining fixed the different types of clamp (Klemmer, Chest-Tube, ECMO). Comparisons of pressures and volumes 30 s after clamping in the two above-mentioned situations (different clamps; different clamping moments) were performed with the Kruskal–Wallis test. By significant *p*-value, post hoc comparisons [two-by-two comparisons to identify which situation differ from the other(s)] with the Mann–Whitney test were carried with α-level correction (like Bonferroni) to take into account the multiple comparison procedure avoiding false positive results. To assess the effects of different clamps as a function of time, we performed a non-parametric one-way repeated-measures analysis of variance (ANOVA) (Friedman test), not followed by post hoc comparisons because we were only interested in trend over time instead of a single time point assessment. To evaluate a possible interaction among different clamps and different clamping moments during the respiratory cycle we performed a two-way ANOVA. The effects of reconnection to the ventilator after a clamping maneuver on airway and driving pressures were assessed only for ETT ID 8. Pressures after reconnection were compared between clamp at end-inspiration and clamp at 50%-end-inspiration by maintaining fixed the type of clamp (Klemmer, Chest-Tube, ECMO) with the Wilcoxon rank sum test. An interesting alternative approach to analyze these multilevel hierarchical data as a whole, would be the exploitation of linear mixed effects models, which are unfortunately not applicable with less than five clusters of data (only three types of clamps; only three clamping moments during the respiratory cycle). All tests were performed two-sided and a *p*-value < 0.05 was considered statistically significant. All analyses were performed with Stata software version 17 (StataCorp LP, College Station, TX, USA).

## Results

### Experimental model

The absolute increase in FRC generated by the application of PEEP (10 cm H_2_O) was 300 ml, considering zero lung volume at FRC-without PEEP. A TV of 360 ml generated an alveolar end-inspiratory pressure of 22 cm H_2_O, DP of 12 cm H_2_O and lung volume of 660 ml (300 ml + 360 ml). With the application of ½TV (180 ml) the end-inspiratory airway pressure was 16 cm H_2_O, DP 6 cm H_2_O and TV 480 ml (300 ml + 180 ml). The ventilator accurately delivered the TV of 360 ml, the only exception being with the ETT ID 6 mm, where the TV at the end of inspiration was slightly higher (368 ml).

ETT don’t show damage after test, the only note can be made for Klemmer clamp that has left superficial scratches.

### Comparison between clamps, ETT sizes and duration of clamping

**Fig. 3:**
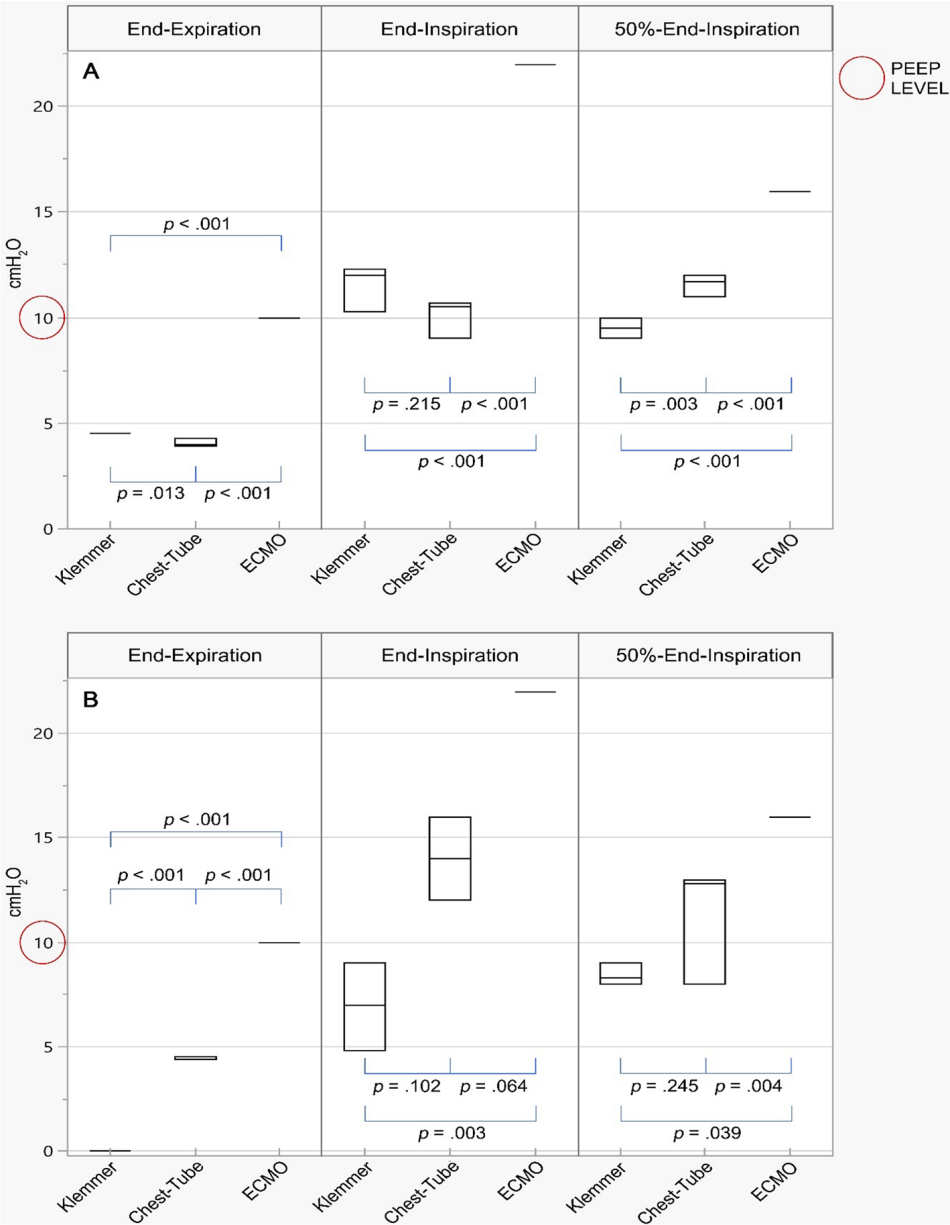
Alveolar pressure distribution 30 s after disconnection from the mechanical ventilator according to type of clamp and to the clamping moment during the respiratory cycle for ETT ID 7 mm (**A**) and 8 mm (**B**). Chest-Tube, tubing clamp forceps 20 cm; ECMO, tubing clamp forceps 18 cm; Klemmer, Rochester-Pèan forceps; end-expiration, clamping during end-expiratory occlusion; end-inspiration, clamping during end-inspiratory occlusion; 50%-end-inspiration, clamping during end-inspiratory occlusion with tidal volume halved

The effects of clamping depended on the type of clamp (Fig. 3), on ETT size and on duration of the clamp itself (Table 1).

**Table 1:** Alveolar pressure and lung volume at 5, 15 and 30 s after disconnection from the mechanical ventilator according to type of clamp and to the clamping moment during the respiratory cycle for ETT ID 6, 7 and 8 mm

ETT	Clamping moment	Clamp	PAW 5 s	PAW 15 s	PAW 30 s	Volume 5 s	Volume 15 s	Volume 30 s
6 mm	End-expiration	Klemmer	10 (10;10)	10 (10;10)	10 (10;10)	300 (300;300)	300 (300;300)	300 (300;300)
		Chest-Tube	10 (10;10)	10 (10;10)	10 (10;10)	300 (300;300)	300 (300:300)	300 (300;300)
		ECMO	10 (10;10)	10 (10;10)	10 (10;10)	300 (300;300)	300 (300;300)	300 (300;300)
	End-inspiration	Klemmer	21,5 (20.8;21,75)	20 (20;20.5)	20 (19.5;20)	645 (622.5;652.5)	624 (612;634)	600 (585;600)
		Chest-Tube	22 (22;22)	22 (22;22)	22 (22;22)	668 (668;668)	668 (668;668)	668 (668;668)
		ECMO	22 (22;22)	22 (22;22)	22 (22;22)	668 (668;668)	668 (668;668)	668 (668;668)
	50%-end-inspiration	Klemmer	16 (16;16)	16 (16;16)	16 (16;16)	480 (480;480)	480 (480;480)	480 (480;480)
		Chest-Tube	16 (16;16)	16 (16;16)	16 (16;16)	480 (480;480)	480 (480;480)	480 (480;480)
		ECMO	16 (16;16)	16 (16;16)	16 (16;16)	480 (480;480)	480 (480;480)	480 (480;480)
7 mm	End-expiration	Klemmer*	8.7 (8.6;8.9)	6.6 (6.6;6.65)	4.5 (4.5;4.5)	256 (255.5;264.5)	197 (196.5;198.5)	136 (135.5;138)
		Chest-Tube*	9 (8.95;9.1)	6.3 (6.25;6.35)	4 (3.95;4.15)	268 (268;272)	188 (186;190.5)	120 (118.5;122.5)
		ECMO	10 (10;10)	10 (10;10)	10 (10;10)	300 (300;300)	300 (300;300)	300 (300;300)
	End-inspiration	Klemmer*	19 (18.8;19.3)	16 (15.5;16.1)	12 (11.15;12.15)	575 (565;580)	470 (460;478)	363 (363;365.5)
		Chest-Tube*	19.2 (19;19.6)	15 (14.5;15.5)	10.5 (9.75;10.6)	579 (569.5;589.5)	455 (437.5;467.5)	317 (293.5;319)
		ECMO	22 (22;22)	22 (22;22)	22 (22;22)	660 (660;660)	660 (660;660)	660 (660;660)
	50%-end-inspiration	Klemmer*	14.5 (14.3–14.95)	12 (11.35;12)	9.5 (9.25;9.75)	438 (429;449.5)	357 (339;358.5)	279 (274.5;289.5)
		Chest-Tube*	15 (15–15)	13,4 (12.7;13.7)	11.7 (11.35;11.85)	451 (450.5;451.5)	402 (381;411)	354 (342;357)
		ECMO	16 (16–16)	16 (16;16)	16 (16;16)	480 (480;480)	480 (480;480)	480 (480;480)
8 mm	End-expiration	Klemmer*	6.8 (6.75;6.8)	2.6 (2.55;2.65)	0 (0;0)	203 (202;204)	77 (76;79)	0 (0;0)
		Chest-tube*	8 (7.85;8.15)	6 (5.9;6)	4.4 (4.4;4.45)	246 (239;247.5)	180 (178.5;180)	131 (130.5;132)
		ECMO	10 (10;10)	10 (10;10)	10 (10;10)	300 (300;300)	300 (300;300)	300 (300;300)
	End-inspiration	Klemmer*	18 (17.5;18.5)	12 (12;12.4)	7 (5.9;8)	540 (525;555)	360 (360;372)	210 (177;240)
		Chest-tube*	20 (19;20)	16.8 (16;17.4)	14 (13;15)	600 (570;605.5)	504 (478.5;526.5)	420 (390;454)
		ECMO	22 (22;22)	22 (22;22)	22 (22;22)	660 (660;660)	660 (660;660)	660 (660;660)
	50%-end-inspiration	Klemmer*	14.5 (13.8;14.75)	11.5 (10.8;11.75)	8.3 (8.15;8.65)	430 (410;440)	345 (322.5;354)	251 (246;260.5)
		Chest-tube*	15 (14;15.5)	13.8 (11.9;13.9)	12.8 (10.4;12.9)	450 (420;464)	419 (359.5;420.5)	384 (313.5;387.5)
		ECMO	16 (16;16)	16 (16;16)	16 (16;16)	480 (480;480)	480 (480;480)	480 (480;480)

The data are shown as median with 1st and 3rd quartiles

Pressure and volume variations as a function of time assessed by Friedman test: *p ≤ 0.001

ETT, endotracheal tube; ID, internal diameter; Paw, airway pressure; end-expiration, clamping during end-expiratory occlusion; end-inspiratory, clamping during end-inspiratory occlusion; 50%-end-Inspiratory, clamping during end-inspiratory occlusion with tidal volume halved; Klemmer, Rochester-Pèan forceps; Chest-Tube, tubing clamp forceps 20 cm; ECMO, tubing clamp forceps 18 cm

With ETT ID 6 mm the three clamps (Klemmer, Chest-Tube, ECMO) showed similar results in preventing the loss of pressure and volume at 30 s post-disconnection.

With an ETT ID 7 and 8 mm only the ECMO clamp maintained stable pressure and volume during disconnection at all observation times. Klemmer and Chest-Tube clamps showed a loss of pressure in all timepoints (5, 15 and 30 s) and at all clamping moment (end-expiration, end-inspiration; 50%-end-inspiration). Klemmer and Chest-Tube showed comparable clamping efficacy in almost all experimental conditions, however the tightness of the Klemmer was strongly influenced by the position of the ETT in the clamp, resulting unstable with frequent and sudden openings.

The volumetric data were consistent with the pressure results.

### Best clamping moment

Clamping at end-inspiration and at 50%-end-inspiration was more effective, compared to clamping at end-expiration for both ETTs ID 7 and 8 mm, and for both clamp Klemmer and Chest-Tube (Table 2). The volumetric data were consistent with the pressure results.

**Table 2:** Alveolar pressure at 5, 15 and 30 s after disconnection from the mechanical ventilator according to type of clamp for ETT ID 7 and 8 mm

ETT	Clamp	Timepoint	Paw			End-expiration vs end-inspiration	End-expiration vs 50%-end-inspiration	End-inspiration vs 50%-end-inspiration
			End-expiration	End-inspiration	50%-end-inspiration	p value	p value	p value
7 mm	Klemmer	5 s	8.7 (8,6;8,9)	19 (18,8;19,3)	14.5 (14,3;15)	< 0.001	< 0.001	< 0.001
		15 s	6.6 (6,6;6,7)	16 (15,5;16,1)	12 (11,35;12)	< 0.001	< 0.001	<0.001
		30 s	4.5 (4,5;4,5)	12 (11,2;12,2)	9.5 (9,25;9,75)	< 0.001	< 0.001	0.033
	Chest-Tube	5 s	9 (8,95;9,1)	19.2 (19;19,6)	15 (15;15)	< 0.001	< 0.001	<0.001
		15 s	6.3 (6,25;6,35)	15 (14,5;15,5)	13.4 (12,7;13,7)	< 0.001	< 0.001	0.099
		30 s	4 (3,95;4,15)	10.5 (9,75;10,6)	11.7 (11,4;11,9)	< 0.001	< 0.001	0.078
8 mm	Klemmer	5 s	6.8 (6,75;6,8)	18 (17,5;18,5)	14.5 (13,8; 14,8)	0.001	0.008	0.241
		15 s	2.6 (2,55:2,65)	12 (12;12,4)	11.5 (10,8;11,8)	0.033	0.021	1
		30 s	0 (0;0)	7 (5,9;8)	8.3 (8,15;8,65)	0.017	0.019	0.498
	Chest-Tube	5 s	8 (7,85;8,15)	20 (19;20)	15 (14;15,5)	< 0.001	0.001	0.001
		15 s	6 (5,9;6)	16.8 (15,95;17,4)	13.8 (11,9;13,9)	< 0.001	0.002	0.002
		30 s	4.4 (4,4;4,45)	14 (13;15)	12.8 (10,4;12,9)	< 0.001	0.019	0.004

The data are shown as median with 1st and 3rd quartiles, endotracheal tube; Paw, airway pressure; end-expiration, clamping during end-expiratory occlusion; end-inspiration, clamping during end-inspiratory occlusion; 50%-end-inspiration, clamping during end-inspiratory occlusion with tidal volume halved; Klemmer, Rochester-Pèan forceps; Chest-Tube, tubing clamp forceps 20 cm. *p-*values are adjusted for take into account multiplicity

### Airway pressure and driving pressure after reconnection

**Fig. 4:**
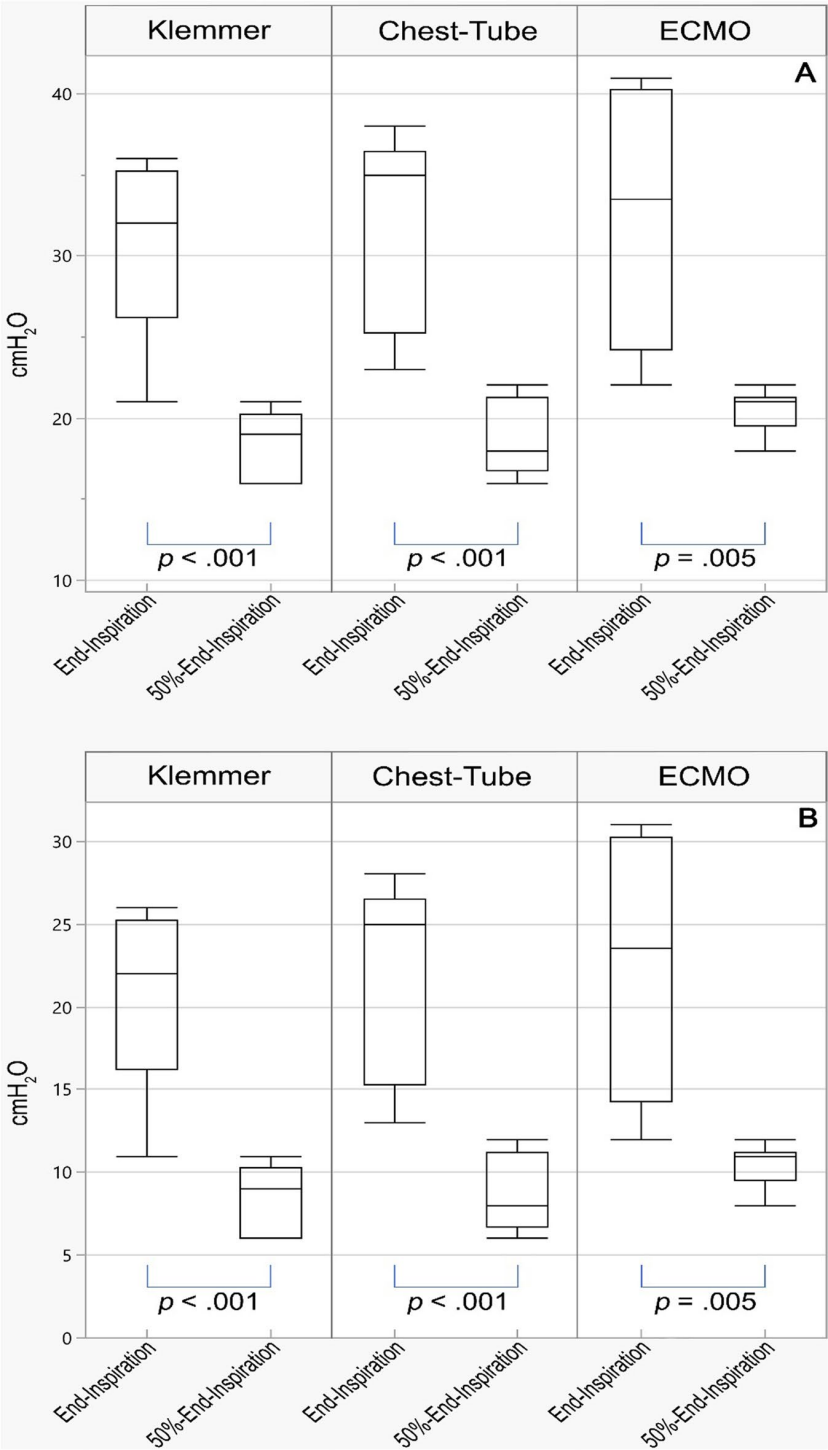
Plateau pressure (**A**) and driving pressure (**B**) after reconnection to the mechanical ventilator after a clamping maneuver at end-inspiration and at end-inspiration with tidal volume halved. Klemmer, Rochester-Pèan forceps; Chest-Tube, tubing clamp forceps 20 cm; ECMO, tubing clamp forceps 18 cm.; end-expiration, clamping during end-expiratory occlusion; end-inspiration, clamping during end-inspiratory occlusion; 50%-end-inspiration, clamping during end-inspiratory occlusion with tidal volume halved. *p*-values are adjusted for take into account multiplicity

End-expiration clamp removal (shortly before insufflation) generated greater pressures and volumes than a removal at the beginning of expiration. The comparison of airway pressures after reconnection to the ventilator between the two clamping moments (at end-inspiration and at 50%-end-inspiration) was statistically significant for all clamps (Klemmer, Chest-Tube, ECMO). Median Pplat on reconnection after occlusion at end-inspiration varied between 32 and 35 cm H_2_O, depending on the type of clamp used, with a range between 22 and 42 cmH_2_O (Fig. 4a). Median DP on reconnection after occlusion at end-inspiration varied between 22 and 25 cm H_2_O, depending on the type of clamp with a range between 11 and 31 cmH_2_O (Fig. 4b).

The three clamps produced similar data for reconnection after occlusion at end-inspiration (*p* = 0.882) and for reconnection after occlusion at 50%-end-inspiration (*p* = 0.179). The volumetric data were consistent with the pressometric results.

## Discussion

In the present study, aiming at assessing the effects of three different types of clamps on ETT of different sizes and at different clamping moments, we found that pressure and volume losses are affected by the type of clamp, the size of the ETT, the duration of clamping and the clamping moment during the respiratory cycle. Moreover, the reconnection to the mechanical ventilator after a clamping maneuver is associated with the development of high airway pressures.

The most effective clamp to prevent significant pressure and volume losses was the ECMO, in keeping with the only previously published study [12]. Klemmer and Chest-Tube performed similarly. The size of the clamp seems decisive for the grip stability, but by itself it does not completely explain what has been observed. In fact, Chest-Tube, despite being the largest tested clamp, it performs worse than ECMO. This may be explained by the pressure exerted by the clamp on the ETT, defined as the ratio between the force, acting perpendicularly on a surface, and the area of the contact surface (*Pressure* = *Force/Surface).* Accordantly, pressure is thus directly proportional to the force and inversely proportional to the contact surface area. The clamp’s size and the operator’s force performing the clamping maneuver determines the *Force,* while the shape/surface of the ETT determines the *Surface*. ECMO has a contact surface of 2 mm, much lower than the Chest-Tube (6 mm). This may explain why ECMO is more effective despite its smaller size. On the other hand, Klemmer has a contact surface of 3.5 mm, which can taste its similar performance to Chest-Tube, despite different size.

The size of the ETT also impacts the effects of clamping on pressure and volume losses, except for the smallest ETT. In fact, for ETT ID 6 mm, the type of clamping and the moment of clamping showed comparable effects. This finding is probably related to the fact that ETT ID 6 mm, being more malleable at compression, the force of the tested clamps is sufficient to produce an effective occlusion of the tube.

Another determining factor for the efficacy of clamping is the duration of clamping itself. For Klemmer and Chest-Tube, pressure and volume losses occurred as early as 5 s after disconnection. The efficacy of ECMO clamp was not affected by the duration of clamping, in keeping with the previous bench study, showing only a minimal decrease in alveolar pressure 30 s after disconnection [12].

The moment of clamping during the respiratory cycle also impacts the efficacy of clamping. Clamping at end-inspiration, as well as, at 50%- end-inspiration, generates, in contrast to clamping at end-expiration, alveolar pressures greater than or equal to PEEP. Statistically significant differences between the effects of clamping at end-inspiration and of clamping at 50%-end-inspiration is to be considered for de-recruitment clinically irrelevant, because Paw remained above 10 cm H_2_O. Consequently, clamping not at end-expiration may be effective in preventing de-recruitment even with less performing clamps.

The effects of reconnection after a clamping maneuver at end-inspiration depend on the de-clamping moment during the respiratory cycle. By clamp removal at the beginning of the expiratory phase, the lung can empty and reach the level of PEEP. Conversely, by clamp removal at end expiration, the new TV adds to already present lung volume generating high Paw and DP.

Perform clamping at end-inspiration could be dangerous for the following reasons:

First, maintaining high lung pressures could reduce right ventricular preload and generate hemodynamic impairment, even more so if the maneuver were to last over time [13–15].

Second, during the reconnection phase, the new tidal volume is added to the remaining lung volume, generating high alveolar pressures on the first insufflation, contributing to a potential risk of alveolar overdistension.

Pplat values > 30 cmH_2_O and DP > 14 cmH_2_O [16, 17] have been associated with an increased mortality in patients with ARDS, although this type of high pressures is applied in instantaneous moments such as recruitment maneuvers [18, 19]. Yet, in order to optimize protective ventilation and prevent ventilator induced lung injury (VILI), it seems better to avoid uncontrolled high alveolar pressures on reconnection. This occurrence can be mitigated using a pressometric ventilation or by setting an adequate maximum safety pressure in VCV. Clamping at 50%-end-inspiration is more effective than clamping at end-expiration and generated safer airway pressures after reconnection to the ventilator.

By halving the TV to perform the maneuver, the risk of lung overdistension is limited. In fact, by halving the TV, even if the clamp is removed at the end of the expiration, the new TV (= 180 ml) is added to the previous one (= 180 ml) thus reaching a lung volume equal to the TV used before the maneuver. Although we found a greater efficacy of clamping at 50%-end-inspiration as compared to end-expiration in maintaining a Paw > 10 cm H_2_O care should be taken in case of leaks.

The extent of air loss generated by a poorly performing clamp can be unpredictable in daily practice and depends on many variables such as the clamp used, the force with which it is tightened and the use of gauze to protect the ETT during clamping.

This study has several limitations. Although ASL 5000 is a very realistic simulator, there are currently no in vivo data (both clinically and in intact animal models) on the efficacy of clamps or on the extent of any pressure loss during clamping. For example, the effects of clamps may differ from ours only considering the thermosensitivity of ETT, which will tend to soften when in contact with the upper respiratory airways. Another confounding factor that could generate differences in terms of increased losses, at the bedside, is the use of one or more gauzes during clamping to protect the ETT. In addition, the number of measurements carried out in the laboratory was modest, and we cannot exclude a greater variability of the data compared to that obtained. However, our study has several strengths. The effects of clamping were assessed for ETT of different size. The clamping moment during the respiratory cycle was not restricted to the end of an expiration. Finally, we evaluated pressure behavior following the reconnection to the ventilator after a clamping maneuver.

## Clinical implication

Frequent twist and sudden openings make Klemmer ineffective and unreliable, and consequently we discourage its use in adult’s ETT clamping.

By using a Chest-Tube clamp, we suggest limiting the duration of disconnection from the ventilator or perform ETT clamping at 50%-end-inspiration. We suggest using ECMO by default at end-expiration.

Even if ETT do not show damage after clamping test, does not possibly exclude that in clinical practice, especially in long-term intubated patients submitted to repeated tube clamping, any damage of the ETT can occur.

## Conclusions

Only ECMO clamp was effective in preventing the loss of PEEP regardless of the ETT size and the duration of the clamping. Clamping at end-inspiration, i.e., at larger lung volumes and pressures than end-expiration, increased the effectiveness of underperforming clamps such as Klemmer and Chest-Tube. However, clamping at end-inspiration is discouraged due to the considerable increase in pressures at the time of reconnection. If less effective clamps are used with clamping at end-inspiration, then halving the tidal volume could prevent alveolar de-recruitment and eliminate the risk of overdistension on the subsequent reconnection Ultimately, for adult ETTs, our findings support the use of ECMO clamp and clamping during end-expiration.

## Data Availability

Authors can confirm that all relevant data are included in the article and/or its supplementary information files.

## Abbreviations

ARDS: Acute respiratory distress syndrome
Chest-Tube: Tubing clamp forceps 20 cm
ECMO: Tubing clamp forceps 18 cm
Klemmer: Rochester-Pèan forceps
Crs: Compliance respiratory system
DP: Driving pressure
End-expiration: Clamping performing during end-expiratory occlusion
End-inspiration: Clamping performing during end-inspiratory occlusion
50%-end-expiration: Clamping performing during end-inspiratory occlusion with half tidal volume
ETT: Endotracheal tube
FRC: Functional residual capacity
ID: Internal diameter
Palv: Alveolar pressure
PAW: Pressure airway
PCV: Pressure controlled ventilation
PEEP: Positive end-expiratory pressure
Pplat: Plateau pressure
RR: Respiratory rate
VCV: Volume controlled ventilation
VILI: Ventilator induced lung injury
Vol: Pulmonary volume
TV: Tidal volume
